# Videogame-related experiences among regular adolescent gamers

**DOI:** 10.1371/journal.pone.0235327

**Published:** 2020-07-10

**Authors:** José Antonio Ponce-Blandón, Inocencio Espejel-Hernández, Macarena Romero-Martín, María de las Mercedes Lomas-Campos, Nerea Jiménez-Picón, Juan Gómez-Salgado

**Affiliations:** 1 Red Cross Nursing University Center, University of Seville, Sevilla, Spain; 2 Nursing, Physiotherapy and Podiatry Faculty, University of Seville, Sevilla, Spain; 3 Faculty of Labour Sciences, Department of Sociology, Social Work and Public Health, University of Huelva, Huelva, Spain; 4 Safety and Health Postgraduate Programme, Espiritu Santo University, Guayaquil, Ecuador; Sapienza - University of Roma, Italy, ITALY

## Abstract

The objective of this study was to identify the videogame-related experiences expressed by regular adolescent gamers and to explore the socio-family factors related to these experiences. A cross-sectional observational and descriptive study was carried out with a convenience sample of regular Spanish videogamers between 16 and 18 years old. To measure the use of videogames for evasion and its negative consequences, the Questionnaire of Videogame-Related Experiences (*Cuestionario de Experiencias Relacionadas con Videojuegos*, CERV) was used and socio-family variables collected, evaluating their relationship with the results of the CERV. A total of 206 adolescents participated, 89.3% men [84.3–93.2] and 17.9% [12.9–23.9] allocating more than 35 hours a week to videogames. The CERV subscale related to the evasive use of videogames (max. = 24 points) obtained a mean value of 11.71 (SD = 3.52) and the mean value for the subscale related to the negative consequences (max. = 27 points) was 7.14 (SD = 3.33). A higher frequency of high values of evasive use (p = .038) and higher scores of this subscale (p = .02) were found in gamers without brothers or sisters. Higher scores and larger numbers of negative consequences were found in gamers who play more than 21 hours a week (p = .032). In conclusion, frequent use of videogames does not seem to be carried out with an evasive purpose, except in the case of absence of siblings. Frequent videogame use has only proven to carry a higher level of negative consequences when playing more than 21 hours a week. No other socio-family variables related to these subscales of the CERV have been identified.

## 1 Introduction

In this new digital era, technology is more and more accessible and, nowadays complex videogames of different genres are available, creating a wide variety. Currently, the users can access games that combine the action and adventure genres with an open and free scenario [1] but the use of Internet is worth mentioning as a current significant element. The ability to play online has opened doors and created new frontiers, allowing to share the experience with dozens, hundreds and thousands of players at the same time [2].

Despite the arguments speculating the negative effect of videogames, a fair share of them can contribute towards cognitive benefits [3] since they improve attention, spatial skills and mental rotation capacities. Additionally, regular gamers are better at filtering irrelevant information around them [4]. It is also argued that videogames can be an excellent tool for problem solving and that they could enhance creativity. Another benefit is the constant balance between frustration, challenge and success [3], adding to the emotional benefit, the increasing capacities in relation to physical activity and education [5].

However, videogames are no longer limited to video consoles in our homes and this can result in prolonged use. This mainly affects a younger age range of the population as they are in a critical stage of developing healthy lifestyles and many of these youngsters do not comply with the recommended practice of physical activity [6]. Together with the abuse of videogames, such as gambling disorders, gaming disorders, Internet use disorders, and excessive smartphone use [7], this could develop into behavioural addictions [8], including those related to handling electronic devices with screens [9] and high inactivity levels, which could cause chronic diseases [6] turning videogames into a factor which affects heath negatively. In fact, some studies have proved that teenagers with internet addiction, including “online gaming”, experience lower quality in their relationships with parents and more individual difficulties [10]. In addition, the increase of Internet use has been positively related to depressive symptoms in adolescents, considering the results of an study conducted in Taiwan [11]. Furthermore, there is another study that has proven that early maturing and thin-weight adolescents are more exposed to spend long hours on Internet, and also, early puberty has been associated with online pornography viewing among men, concerning the use of new technologies in adolescents [12].

In recent years, many studies on videogames and their effects on the younger population have been conducted [13–17]. This evidence shows that it is important to determine the extent to which videogame use can cause harm in terms of family relations, academic performance and maturation during adolescence. Perhaps one of the best indicators of the increasing interest is the recent inclusion of the online game disorder in the third section of the DSM-5 manual [18].

Furthermore, during the specific evolutionary phase of adolescence, characterized by different physical, biological, psychological and social changes [19, 20], individuals become especially vulnerable to suffer some problems specifically identified as eating disorders [21, 22] or addictive disorders [23].

There are also studies that have explored the relationships between body mass index (BMI) and adolescent lifestyle behaviours, finding a link between sedentary behaviours, such as using computer (non-school related) or playing video or computer games, with BMI [24]. Sedentary behaviour or time spent in video games was associated with increases in BMI [25] with various levels of obesity [26], not only in adolescents, but also in preschool children [27].

The interest to analyse socio-family factors, related to the use of video games in adolescents, has also been frequently shown in recent research [28], including topics like developing and validating scales to measure the influence of family factors on physical activity and time spent on “screen media practices” [29]. The role of the family has also been highlighted in some works as relevant in the appearance of problems such as the internet gaming disorder [30, 31].

Using a great variety of different scales to measure addiction to videogames hinders estimates of prevalence and potential effects, since this variability does not allow for a standardized measurement [17]. Whereas the tools for the general population have advanced considerably in the last few years, the current work with children and adolescents has few specific assessment instruments. However, some interesting scales related to work with children and adolescents have been developed in Spain. In this respect, Pedrero-Pérez et al. [32] developed a screening scale based on Ewing’s CAGE scale (a scale for detecting alcohol abuse) of 1984. Tejeiro-Salguero and Bersabé-Morán [33] built the “Problem Video Game Playing Questionnaire” (later known as “PVP”), Chóliz and Marco [34] built the “Video Game Dependence Test” questionnaire and, finally, Labrador, Villadangos, Crespo and Becoña [35] developed a measuring scale for the problematic use of new technologies, all based on the criteria established in the DSM-4.

One of the most interesting and original tools is the Questionnaire of Videogame-Related Experiences (*Cuestionario de Experiencias Relacionadas con Videojuegos*, CERV), validated in Spain in 2014 [13]. Although it was designed as a specific tool to value evasion and the negative consequences of videogames in young people and adolescents, it has not been used very frequently. It is a very complete tool because it integrates the Questionnaire of Internet-related experiences (*Cuestionario de experiencias relacionadas con Internet*, CERI) and the Questionnaire of mobile phone-related experiences (*Cuestionario de experiencias relacionadas con el móvil*, CERM) into a single version [36].

The CERV questionnaire has 17 items on denial, concern, negative effects, tolerance increase, loss of control, reduction of activities, evasion and craving for playing videogames. Such items were built from the sub-scales of intra- and inter-personal conflicts of the CERI questionnaire and from the conflict scale of the CERM questionnaire, all made up by scales of four point Likert-type answers. It is validated by means of a sample of more than 5,500 students of compulsory high school [13], with Cronbach's alpha coefficients of 0.869 for the sub-scale of negative consequences and of 0.861 for its evasion counterpart. New studies using this tool applied to populations different from that of their validation would be required, particularly in young people and adolescents who make frequent use of videogames, since the usage of the tool has only been explored in the young and adolescent population in general, with no distinction between frequent or infrequent use of videogames.

It is for all these reasons previously outlined that it is considered convenient to conduct an exploratory study on the possible effects of using videogames on the different aspects of everyday life in a population of regular adolescent videogame users, with the Questionnaire of Videogame-Related Experiences (CERV) as a tool. The objective of the present study is to identify the population of gamers in these age ranges who abusively use videogames, to understand the extent of its use for evasion and to determine the negative consequences of videogames in these individuals, apart from the socio-family factors which could be related with all these consequences.

## 2 Material and methods

A cross-sectional, observational and descriptive study was conducted to assess the videogame-related experiences in a group of young regular videogamers aged 16 to 18 years old in the Spanish national territory, exploring some hypotheses to identify sociodemographic variables that could be related to the abusive use of videogames, their use for evasion and the negative experiences related to their use.

### 2.1 Sample and procedure

The study population was made up of regular videogamers residing in Spain, registered on the «Twitter» social network, and aged 16 to 18 years old. A convenience sampling was performed, accessing the study population through a call to the users following the «@GamerEnfurecido» profile, and completion of a specific questionnaire circulated through that profile. This account, with over 30,000 users, specializes in the review and opinion of the ongoing events in the videogame world, and it is one of the benchmarck profiles in Spain among the users of the sector; this is why people following it in this social network are regular gamers searching for current information on videogames. The following exclusion criteria were established for participating in the sample:

- Being less than 16 or over 18 years old.
- Filling in the questionnaire outside the deadlines.
- Residing abroad.
- Giving incoherent answers.
- Not complying with the mandatory items established in the questionnaire.

### 2.2 Measures

Sociodemographic variables that were collected, used for the descriptive analysis and as independent variables in the exploratory hypothesis, were the following: age, gender, schooling, province of residence, size of the municipality of residence, family situation of the participant (living or not with both parents or with one of them, presence of siblings), and frequency of videogame use expressed in playing hours a week. The BMI (Body Mass Index) was also calculated from the weight and height of the participants.

Simultaneously, data from the assessment of evasion and of the negative consequences of videogames in young people and adolescents through the CERV tool was collected [13], with 17 items or questions on denial, concern, negative effects, tolerance increase, loss of control, reduction of activities, evasion and craving for playing videogames, using a four point Likert type scale. These items are structured in two categories: one on the use of videogames for evasion and the other on the negative consequences of videogame use. The values in the scales of each of these categories were also considered as dependent variables of the exploratory hypothesis.

The creators of the authorization of use scale were asked to adapt it to a digital format, and the process was performed by means of the «Google Forms®» platform. All the scale items were kept as mandatory questions. Subsequently, question fields were added for the socio-family variables, also mandatory, as well as weight and height. To ensure that the same user could not fill in the questionnaire more than one time, Google Forms tool registers the network ID. With this information the researchers excluded the network ID repeated, which would indicate that the form had been completed twice from the same device or user.

An access link was obtained for its distribution and 140 character «tweets» were scheduled asking the users following the «@GamerEnfurecido» account to fill in the questionnaire from 5/20/2016 to 7/20/2016. Once the deadline was over, the results were exported into a spreadsheet, for a subsequent debugging of the data by applying the pre-established exclusion criteria.

### 2.3 Data analysis

Data analysis was done with the «Epi Info version 7®» and «IBM SPSS version 24®» tools. For the descriptive analysis of the qualitative variables the frequencies were calculated with a 95% confidence interval, whereas for the quantitative variables a numerical summary was done by calculating the centralization and dispersion measures.

With reference to the contrast of the exploratory hypothesis set out, bivariate analysis were performed; to this end, the results of each proportion of the CERV scale were re-coded by assigning them values from 0 to 3 points and by performing a summation based on the categories they belonged to. This result obtained, the results of each subscale were arranged in two groups, «high value» and «low value», depending on whether the numerical score was below or over the mean value of the subscale’s total score.

In the same way, the independent variables which presented more than two categories were dichotomized; and these hypothesis were contrasted through the Pearson’s Chi-square test, except for those hypothesis in which the Fisher’s exact test had to be used due to the existence of unexpected values below 5 in the contingency table. The risk ratios were also calculated, with their confidence interval set at 95%. Thus both, independent variable and the result variable, were dichotomized since in this way they better represent the “positive” or “negative” status of each variable and can facilitate the interpretation of the coefficients of the statistical model, as established by some authors [37]. Another important reason is related to the assumptions of a statistical model and because the categorization of continuous variables allows researchers to avoid the strong assumptions required by the applied model about the relationship between variables and risk measurement, without losing too much information in this case, following Altman et al. [38]. Because the linearity assumption is not a requirement when using categorical variables, these authors prefer to use methods that require the minimum of assumptions. The outpoints were established in the means and/or medians as indicated by Maxwell and Delaney [39] as recommended for the contrast of two continuous independent variables.

However, to avoid the bias of losing of relevant information that this recategorization of the variables may induce, and in order to check the consistency of the results a second contrast of the hypothesis was performed, keeping the numerical values in the CERV scale subscales, and using the parametric Student’s T test for the comparison of mean values or the Mann-Whitney’s U test, in case the parametricity criteria were not met, for which the Kolmogorov–Smirnov tests were previously used in order to check the normality of the numerical variables of the CERV scale.

The significance level for all the hypothesis contrasts was established in a p-value below 0.05.

### 2.4 Ethic procedures

The privacy of the participating adolescents was preserved so that the questionnaire was completely anonymous and it began with information about the study, including an informed consent for their participation. In accordance with Spanish legislation on protection of personal data, which establishes that data of those over fourteen years old may be processed with their own consent, the access of minors to the twitter account and, therefore, the questionnaire, was open to the option and authorization of participants by themselves.

On the other hand, the study was authorized by the Research Ethics Committee of Spanish Red Cross Nursing School of the University of Seville, the centre from which the researchers carried out the study. The need for parental consent was specifically waived by this ethics committee, through the authorization document.

## 3 Results

After distributing the questionnaire, 270 final answers were obtained. After applying the exclusion criteria, the sample was eventually composed of 206 participants with valid answers. With regard to the sociodemographic description of the sample, 89.3% of the participants were men ([95% CI 84.3–93.2] n = 184) and the mean age was 17.2 years old ([95% CI 17.0–17.3] SD = 0.81). The region from where the greatest number of participants came from was Andalusia (South region in Spain), with 20.7% ([95% CI 15.3–27.0] n = 41), and 43,2% of the sample resided in cities with over 100,000 inhabitants ([95% CI 36.3–50.2] n = 89). Regarding schooling, 57,7% were high school students ([95% CI 50.7–64.6 n = 119), 20.4% were university students ([95% CI 15.1–26.5] n = 42) and the rest were in other levels of study or not studying. With respect to their family situation, 76.2% of the participants lived with their parents, the couple living together, ([95% CI 69.8–81.8] n = 157) and 80.1% of them lived with at least one brother/sister ([95% CI 73.9–85.3] n = 165).

In relation to playing videogames, it is to be noted that 17.9% devoted more than 35 hours a week to playing videogames ([95% CI 12.9–23.9] n = 37). The distribution of the participants based on the number of weekly hours they devoted to videogames is reflected in Table 1.

**Table 1:** Number of hours the participants devote to videogames.

	Frequency	Percentage	Accrued Percentage	95% Confidence Interval
**Less than 7 hours/week**	8	3.8	3.8	1.7–7.5
**Between 7 and 14 hours/week**	41	19.9	23.7	14.7–26.0
**Between 15 and 21 hours/week**	37	17.9	41.6	12.9–23.9
**Between 22 and 28 hours/week**	48	23.3	64.9	17.7–29.7
**Between 29 and 35 hours/week**	35	17.0	81.9	12.1–22.8
**More than 35 hours/week**	37	17.9	100.00	12.9–23.9
**Total**	206	100.0		100.0–100.0

With respect to the BMI calculated from the height and weight informed by the participants, the mean of the group was 22.4 kg/m^2^ ([95% CI 21.7–23.2] SD = 3.6), the «normal weight» situation being the most common, with 71.4% of the participants in this BMI category (95% CI 67.1–80.5] n = 125). The distribution of the participants in the different BMI categories can be seen in Table 2.

**Table 2:** Categorized BMI of the participants.

	Frequency	Percentage	Accrued Percentage	95% Confidence Interval
**Underweight (< 18.5 kg/m**^**2**^**)**	16	9.1	9.1	6.7–13.4
**Normal weight (18.5–25 kg/m**^**2**^**)**	125	71.4	80.5	67.1–80.5
**Overweight (25–30 kg/m**^**2**^**)**	23	13.1	93.6	8.5–19.0
**Obesity (> 30 kg/m**^**2**^**)**	11	6.3	100.0	3.2–10.9
**Total**	175	100		100.0–100.0

In relation to the results of the answers to the CERV, 54.8% stated they «almost always» used videogames as a distraction (95% CI [47.8–61.7] n = 113) and 48.0% also expressed that, «almost always», time passes by unnoticed to them when they play videogames. The mean summation value of the answers re-coded to numerical values in the first subscale, related to the evasive use of videogames, was 11.71 ([95% CI 10.9–12.2] SD = 3.52) whereas, for the second subscale, related to the negative consequences of videogame use, the mean summation value of all of the participants’ answers was 7.14 ([95% CI 6.5–8.0] SD = 3.33). The descriptive detail of the frequencies obtained in the answers to the items of the two subscales of the CERV, as well as the mean numerical and summation values, can be seen in Table 3.

**Table 3:** Frequencies of answers in the items of the CERV. Mean and summation values by subscales of the scale.

**Subscale 1 items: «Evasive use of videogames»**	**Never/Almost never**	**Sometimes**	**Many times**	**Almost always**	**Mean value**
Item 1:«*¿To what extent do you feel any concern with themes related to videogames*?»	n = 23	n = 49	n = 74	n = 60	1.83
	11.1%	23.8%	35.9%	29.1%	
	[7.2–16.3]	[11.1–30.2]	[29.3–42.8]	[23.0–35.8]	SD = .97
Item 2:«*¿Do you use videogames as a distraction when you get bored*?»	n = 0	n = 21	n = 72	n = 113	2.44
	0.0%	10.2%	34.9%	54.8%	
	[.0-.0]	[6.4–15.1]	[28.4–41.9]	[47.8–61.7]	SD = .67
Item 3:«*¿How often do you leave what you are doing to spend more time playing videogames*?»	n = 34	n = 110	n = 52	n = 10	1.18
	16.5%	53.4%	25.2%	4.8%	
	[11.7–22.3]	[46.3–60.3]	[19.4–31.7]	[2.3–8.7]	SD = .76
Item 8:«*¿Does playing videogames help you when you have problems*?»	n = 18	n = 41	n = 80	n = 63	1.91
	8.7%	21.8%	38.8%	30.5%	
	[5.2–13.4]	[16.4–28.1]	[32.1–45.8]	[24.3–37.3]	SD = .93
Item 10:«*¿Do you think that life without videogames is boring*, *empty and sad*?»	n = 42	n = 72	n = 56	n = 36	1.41
	20.4%	34.9%	27.1%	17.5%	
	[15.1–26.5]	[28.4–41.9]	[21.2–33.8]	[12.5–23.3]	SD = 1.00
Item 11:«*¿Do you get angry or upset when someone bothers you while you are playing some videogame*?»	n = 43	n = 101	n = 42	n = 20	1.18
	20.8%	49.0%	20.4%	9.7%	
	[15.5–27.0]	[42.0–56.0]	[15.1–26.5]	[6.0–14.6]	SD = .87
Item 15:«*¿Do you underestimate the time you have been playing videogames*?»	n = 59	n = 86	n = 36	n = 25	1.13
	28.6%	41.7%	17.5%	12.1%	
	[22.5–35.3]	[34.9–48.8]	[12.5–23.3]	[8.0–17.4]	SD = .96
Item 16:«*¿Do you refrain from going out with your friends to spend more time playing videogames*?»	n = 116	n = 68	n = 10	n = 12	0.60
	56.3%	33.0%	4.8%	5.8%	
	[49.2–63.2]	[26.6–39.8]	[2.3–8.7]	[3.0–9.9]	SD = .83
**Summation value for subscale 1**					**11.71**
					**SD = 3.52**
					**[10.9–12.2]**
**Subscale 2 items: «Negative consequences of videogame use»**	**Never/Almost never**	**Sometimes**	**Many times**	**Almost always**	**Mean value**
Item 4:«*¿Have your friends or relatives criticized you for investing too much time and money in videogames*, *or have they told you that you have a problem*, *although you think it is not true*?»	n = 47	n = 70	n = 62	n = 27	1.33
	22.8%	33.9%	30.1%	13.1%	
	[17.2–29.1]	[27.5–40.9]	[23.9–36.9]	[8.8–18.5]	SD = .97
Item 5:«*¿Have you been at risk of losing an important relationship*, *a job or an academic opportunity for using videogames*?»	n = 175	n = 24	n = 5	n = 2	0.19
	84.9%	11.6%	2.4%	0.9%	
	[79.3–89.5]	[7.6–16.8]	[.8–5.5]	[0.1–3.4]	SD = .51
Item 6:«*¿Do you think your academic performance has been affected negatively by your videogame use*?»	n = 111	n = 70	n = 20	n = 5	0.60
	53.8%	33.9%	9.7%	2.4%	
	[46.8–60.8]	[27.5–40.9]	[6.0–14.6]	[0.8–5.5]	SD = .76
Item 7:«*¿Do you lie to your relatives or friends about the frequency and time you invest in videogames*?»	n = 149	n = 42	n = 7	n = 8	0.38
	72.3%	20.4%	3.4%	3.9%	
	[65.7–78.3]	[15.1–26.5]	[1.4–6.8]	[1.7–7.5]	SD = .73
Item 9:«*¿How often do you block the upsetting thoughts about your life and you substitute them for the nice ones of videogames*?»	n = 37	n = 73	n = 57	n = 39	1.47
	17.9%	35.4%	27.6%	18.9%	
	[12.9–23.9]	[28.9–42.4]	[21.7–34.3]	[13.8–24.9]	SD = .99
Item 12:«*¿Do you suffer from sleep disorders due to some aspects related to videogames*?»	n = 165	n = 29	n = 10	n = 2	0.26
	80.1%	14.0%	4.8%	09%	
	[73.9–85.3]	[9.6–19.6]	[2.3–8.7]	[.1–3.4]	SD = .59
Item 13:«*¿Do you feel unrest or worries when you are not playing videogames*?»	n = 159	n = 45	n = 2	n = 0	0.23
	77.2%	21.8%	0.9%	0.0%	
	[70.8–82.7]	[16.4–28.1]	[.0-.0]	[.0-.0]	SD = .44
Item 14:«*¿Do you feel the need to invest more and more time in videogames to feel satisfied*?»	n = 152	n = 40	n = 8	n = 6	0.35
	73.8%	19.4%	3.8%	2.9%	
	[67.2–79.6]	[14.2–25.5]	[1.7–7.5]	[1.0–6.2]	SD = .69
Item 17:«*¿Does time passes by unnoticed to you when you use videogames*?*»*	n = 2	n = 37	n = 68	n = 99	2.28
	0.9%	17.9%	33.0%	48.0%	
	[.1–3.4]	[12.9–23.9]	[26.6–39.8]	[41.0–55.1]	SD = .78
**Summation value for subscale 2**					**7.14**
					**SD = 3.33**
					**[6.50–8.03]**

Regarding the analysis of the exploratory hypothesis, for the first subscale, referring to the evasive use of videogames, 53.9% of the participants were in the high values of this subscale in the CERV scale (n = 111), in opposition to 46.2%, who were in the low values of the subscale (n = 95). The absence of siblings was related to higher values of the scale in this subscale, both when categorizing the variables (p = .038) and when comparing the summation values (p = .002). No statistically significant differences were found in the positioning of the participants in the high and low values of the scale, when separating by the gender, size of the city of residence, family situation, BMI category and weekly frequency of videogame use variables. No statistically significant difference was either found when comparing the mean summation values of this subscale in each subgroup for any of these last variables mentioned. The details of these results can be seen in Table 4.

**Table 4:** Contrast of the exploratory hypothesis related to subscale 1: «Evasive use of videogames».

Contrasted variables	Categories	Frequency % (n)	Relative risk [95% CI]	Chi-2 value/ Fisher’s exact test (DoF)	p-value	Frequency (n)	Student’s t value (DoF)	p-value
		«High value»: 53.9% (n = 111)				Mean summation value (SD)		
		«Low value»: 46.2% (n = 95)						
**Gender**	Men	High value: 53.8% (n = 99)	.98 [.65–1.47]	0.004 (1)	.947	n = 184 11.73 (3.3)	0.252 (204)	.802
		Low value: 46.2% (n = 85)						
	Women	High value: 54.5% (n = 12)				n = 22 11.54 (2.60)		
		Low value: 45.5% (n = 10)						
**Type of city**	Municipality >100,000 inhab.	High value: 48.3% (n = 43)	0.83 [.63–1.08]	1.955 (1)	.162	n = 89 11.71 (3.75)	0.019 (204)	.894
		Low value: 51.7% (n = 46)						
	Municipality ≤100,000 inhab.	High value: 58.1% (n = 68)				n = 117 11.70 (3.42)		
		Low value: 41.9% (n = 49)						
**Family situation**	Living with a parent or living alone	High value: 46.9% (n = 23)	1.19 [.85–1.65]	1.247 (1)	.264	n = 49 11.24 (3.22)	1.087 (204)	.280
		Low value: 53.1% (n = 26)						
	Living with both parents	High value: 56.1% (n = 88)				n = 157 11.85 (3.52)		
		Low value: 43.9% (n = 69)						
**Siblings**	Presence of siblings	High value: 50.3% (n = 83)	1.35 [1.04–1.75]	4.276 (1)	.038*	n = 165 Mean = 11.33 (3.21)	3.130 (204)	.002*
		Low value: 49.7% (n = 82)						
	No siblings	High value: 68.3% (n = 28)				n = 41 Mean = 13.21 (3.51)		
		Low value: 31.7% (n = 13)						
**BMI category**	Overweight/Obesity	High value: 52.9% (n = 18)	0.92 [.64–1.32]	0.175 (1)	.675	n = 34 Mean = 12.47 (3.62)	1.37 (173)	.172
		Low value: 47.0% (n = 16)						
	Underweight/Normal weight	High value: 48.9% (n = 69)				n = 141 Mean = 11.56 (3.41)		
		Low value: 51.0% (n = 72)						
**Frequency of videogame use**	>21 hours/week	High value: 55.0% (n = 66)	1.05 [.8–1.36]	.144 (1)	.704	n = 120 Mean = 11.71 (2.75)	.014 (204)	.988
		Low value: 45.0% (n = 54)						
	≤21 hours/week	High value: 52.3% (n = 45)				n = 86 Mean = 11.70 (3.18)		
		Low value: 47.7% (n = 41)						

(DoF): Degrees of Freedom.

*: Statistically significant difference.

Similarly, for the second subscale of the CERV, referring to the negative consequences of videogame use, 40.8% of the participants were in the high values of this subscale of the scale (n = 84), in opposition to 59.2%, who were in the low values of the subscale (n = 122). No statistically significant differences were found in the positioning of the participants in the high and low values of this subscale, when contrasting with all the variables explored, namely: gender, size of the city of residence, family situation, presence of siblings, BMI category, and weekly frequency of videogame use. However, when comparing the mean summation values of this subscale, higher mean values were found when separating the participants based on the weekly number of hours devoted to playing videogames (p = .032), although no statistically significant differences were found for the rest of the variables. These results are reproduced in detail in Table 5.

**Table 5:** Contrast of the exploratory hypothesis related to subscale 2: «Negative consequences of videogame use».

Contrasted variables	Categories	Frequency % (n)	Relative risk [95% CI]	Chi-2 value/ Fisher’s exact test (DoF)	p-value	Frequency (n)	Student’s t value (DoF)	p-value
		«High value»: 40.8% (n = 84)						
						Mean summation value (SD)		
		«Low value»: 59.2% (n = 122)						
**Gender**	Men	High value: 40.8% (n = 75)	0.99 [.58–1.69]	0.000 (1)	.989	n = 184 Mean = 7.15 (3.21)	.189 (204)	.851
		Low value: 59.2% (n = 109)						
	Women	High value: 40.9% (n = 9)				n = 22 Mean = 7.04 (2.21)		
		Low value: 59.1% (n = 13)						
**Type of city**	Municipality >100,000 inhab.	High value: 40.4% (n = 36)	0.98 [.70–1.37]	0.006 (1)	.933	n = 89 Mean = 7.08 (3.23)	.206 (204)	.836
		Low value: 59.6% (n = 53)						
	Municipality ≤100,000 inhab.	High value: 41.0% (n = 48)				n = 117 Mean = 7.18 (3.62)		
		Low value: 59.0% (n = 69)						
**Family situation**	Living with a parent or living alone	High value: 44.9% (n = 22)	0.87 [.61–1.26]	.452 (1)	.501	n = 49 Mean = 7.51 (3.89)	-.842 (204)	.402
		Low value: 55.1% (n = 27)						
	Living with both parents	High value: 39.5% (n = 62)				n = 157 Mean = 7.03 (3.22)		
		Low value: 60.5% (n = 95)						
**Siblings**	Presence of siblings	High value: 40.0% (n = 66)	1.09 [.74–1.62]	.207 (1)	.649	n = 165 Mean = 7.15 (3.38)	-.130 (204)	.896
		Low value: 60.0% (n = 99)						
	No siblings	High value: 43.9% (n = 18)				n = 41 Mean = 7.09 (3.45)		
		Low value: 56.1% (n = 23)						
**BMI category**	Overweight/Obesity	High value: 47.0% (n = 16)	.78 [.51–1.18]	1.194 (1)	.274	n = 34 Mean = 7.38 (3.17)	.72 (173)	.475
		Low value: 52.9% (n = 18)						
	Underweight/Normal weight	High value: 36.8% (n = 52)				n = 141 Mean = 6.92 (3.41)		
		Low value: 63.1% (n = 89)						
**Frequency of videogame use**	>21 hours/week	High value: 42.5% (n = 51)	1.10 [.78–1.55]	.353 (1)	.552	n = 120 Mean = 7.55 (2.68)	2.153 (204)	.032*
		Low value: 57.5% (n = 69)						
	≤21 hours/week	High value: 38.4% (n = 33)				n = 86 Mean = 6.58 (3.22)		
		Low value: 61.6% (n = 53)						

(DoF): Degrees of Freedom.

*: Statistically significant difference.

## 4 Discussion

The study tried to identify the population of adolescents gamers who abusively use videogames, so, as an initial characterization, the profile of the video gamer participating in this study corresponds to a 17 year old boy, high school student, living in a city with more than 100,000 inhabitants, with brothers/sisters, living with his parents and spending between 3 and 4 hours a day playing videogames (between 21 and 28 hours a week). Despite their frequent sedentary habits, the majority of these videogame users have normal weights according to the BMI values.

Nevertheless, it is necessary to reflect that, as this is a convenience sampling, the results cannot be extrapolated to the general population of all the videogame users, a fact that limits its external validity. Despite all of the aforementioned, the sample obtained through a reference profile from a high impact social network in the videogame users sector, is of much interest since the majority of the studies conducted on this theme have explored the videogame-related experiences in the general population of adolescents. This isone of the few studies which approach the research problem by studying populations of adolescents that specifically play videogames very often.

Similarly, regarding the videogame-related experiences, some biases may exist related to the fact that the individuals are linked to a regular use of videogames, so these positive experiences could be conditioned by a positive predisposition.

It is also necessary to discuss, methodologically, the possibility of losing the information and the power of statistical analyses when recategorizing continuous variables [40]. Some authors suggest that there may be underestimation or overestimation biases about the association [39]. However, it seems that these biases are much more probable when analyses are based on multiple linear regression models or logistic regression models are applicable [41], situations that are not applicable to our study. By this way, taking into account that the categorization of continuous variables has allowed researchers to avoid the strong assumptions required by these models about the relationship between variables and the measurement of risk and considering that “Likert” scale of responses of each item was only 4 points scale, it does not seem likely that too much information has been lost to bias the results [38].

Nevertheless, and in spite of all these limitations, the results have been successful at showing some important characteristics can be found in regular videogamers of these age ranges, considering that high-interest sociodemographic variables have been included in the study defined and categorized in reference texts [42–44].

With respect to the gender of the participants, the expected results have been obtained, supported by other similar studies [45, 46], as the frequency of boys who participated in this study represented an overwhelming majority. Although the reality is that young and teenage boys play more videogames than women [47], evasion use and its negative consequences have not been related to gender.

Users between 16 and 17 years old are the ones who seem to play videogames on video consoles with a higher frequency [48]. This justifies the choice of the age range of the sample used in this study, with a majority of participants in high school, thus configuring the classical course of the education system in this age range [45]. Nevertheless, similar research studies exist which propose studying videogame-related usage and experiences even at younger ages, like in Elementary or Secondary School students [49] or in populations from 6 to 15 years old [50], which, although obtaining similar results in relation to the profile of the regular videogame user, suggest the exploration of this problem in other age ranges.

Our study also tried to understand the extent of its use for evasion and to determine the negative consequences of videogames in adolescents, apart from the socio-family factors which could be related with all these consequences. Although our results are not entirely comparable with the results obtained in the research regarding the validation of CERV scale [13]. However, the results indicated that there was a higher prevalence of problematic use among men and also that problematic use decreased with age, in this case not coinciding with our findings. Although the sample was much broader, it had a different nature, since it did not only include adolescent habitual players, and the object of that study was more focused on the tool validation and not on the analysis of the relationships of these factors itself. In fact, variables that were not essential for our study objectives were not collected, such as the time dedicated to video games, the BMI or the rest of the socio-family variables.

Regarding the family situation, no relation was found to evasive use or with the negative consequences of videogames. In relation to the presence of siblings, the situation of the majority was that of videogame users with siblings in their family setting. The interest of this variable might indicate that firstborns who are regular videogame users instil that leisure activity to the younger siblings, thus exerting an influence on them and, although no relation has been found between the negative consequences of videogame use and this variable, a very consistent relation was indeed found between the variable and the evasive use of videogames, which increases when there are no siblings. This might be explained by the preferences shown in some studies, particularly of the boys (who constitute the majority profile of videogamers), to play their favourite videogames with their siblings [51], so that those lacking this family figure may use videogames in a more evasive way.

Bearing in mind that videogames are considered a sedentary habit, higher overweight or obesity figures might be expected in the participants and it draws our attention that no statistically significant differences were found between overweight or obesity and the negative consequences or the evasive use of videogames, although this finding is also supported by other similar studies [46]. However, in the study by Chacón-Cuberos et al. [52] significant relationships were found between a lower adherence to the Mediterranean diet in the university population and a high frequency of videogame use, a higher number of videogames played and a more problematic videogame use.

The high general frequency of videogame use is to be highlighted, which is an expected results given the profile of the followers of this «Twitter» account, primarily made up of regular videogamers who use this social network to actively search for information on news and comments related to the videogames. Although no studies were found addressing similar populations and, thus hindering comparisons, some studies assert that it is frequent in regular videogamers to play less than 11 hours a week [53]. This does not agree with the findings of this study since, as already verified, the most frequent habit among these users is to play between 21 and 28 hours a week, which demonstrates the strong user profile so common in the sample studied. Other works like meta-analysis developed by Liu et al. [54] who analysed 16 different observational studies in populations of 5 to 18 years, not only with video games, watching TV or connected to the computer, but studying also the relationship between the screen times and depressive symptoms. Also, it is important to mention the study by Hale & Guan [55] who conducted a systematic review of 67 different studies on the relationships between screen hours and sleep in children and adolescents. None of the researches included game usage frequencies as high as those found in our work.

Referring to the measurements of the videogame-related experiences made with the CERV, the global numerical result of the two subscales of the questionnaire presented low values. A priori, this result turns out to be surprising, since the subscales’ scores were expected to be higher because this is a collective group of very regular videogamers. However, the most relevant studies which used this same tool [13, 52] did not find large percentages of users with problems related to videogame use or who used them evasively, in accordance with this paper. But the reality is that the samples from these studies were broader and were made up of university students. Then, compared to adolescents from the general population, these “gamers” do not score higher on the subscales of the CERV measure. Even so, the relationship exhibited between a disproportionate frequency of videogame use (more than 21 hours a week) and the negative consequences of their use stands out, a frequency which, although not confirmed in all the statistical tests performed, is a valid result coherent with what was found in other papers, which link the number of hours children and adolescents spend at the computer with negative social and personal consequences [56, 57].

In any case, in consonance with these results and with the sometimes unfairly apocalyptic vision of the negative effects of videogame use, we must continue investigating the theme in view of the results of some other studies conducted in Spain [58], which are not conclusive as to the negative effects of the different uses of videogames and other technologies; although, to the contrary, there are also studies which have proven the effect that videogames with violent content seem to influence aggressive reactions in some adolescents [59].

In summary, this experience allows us to assert that the questionnaire of videogame-related experiences seem to be a useful tool to measure some of the most common negative consequences of abusing this way of digital leisure, as well as its use to evade from everyday problems.

## 5 Conclusions

The results are not completely conclusive to support the idea that young regular users of videogames use them to evade from isolation or from their family problems. They also do not conclusively support the idea that the family situation may exert any influence on the negative consequences of videogame use, thus discarding the idea that they constitute a necessarily harmful habit acquired during conflictive situations in order to escape from them. However there does seem to exist certain evidence that spending a disproportionate time playing videogames can have negative consequences. We must direct education efforts on healthy habits and physical activity to the regular adolescent gamers, being mindful that videogames and physical activity should not be considered as opposing activities, for they can be alternated so as to be complementary.

Because of the results obtained, very valuable information has been acquired on regular adolescent videogame users, information which will pave the way for new lines of research, using broader and more representative samples, as it is necessary to characterize these habits so that education and health professionals can promote health in the community in face of the abusive use of this form of digital leisure.

## Supporting information

S1 Data English questionnaire CVM. (XLSX)
